# Factor inhibiting HIF (FIH-1) promotes renal cancer cell survival by protecting cells from HIF-1*α*-mediated apoptosis

**DOI:** 10.1038/bjc.2011.73

**Published:** 2011-03-08

**Authors:** M N Khan, T Bhattacharyya, P Andrikopoulos, M A Esteban, R Barod, T Connor, M Ashcroft, P H Maxwell, S Kiriakidis

**Affiliations:** 1 Centre for Cell Signalling and Molecular Genetics, Rayne Institute, University College London, London, WC1E 6JF, UK; 2 Stem Cell and Cancer Biology Group, Key Laboratory of Regenerative Biology, South China Institute for Stem Cell Biology and Regenerative Medicine, Guangzhou Institutes of Biomedicine and Health, Chinese Academy of Sciences, Guangzhou 510663, China

**Keywords:** clear cell, HIF-1*α*, FIH-1, HIF-2*α*, apoptosis, renal carcinoma

## Abstract

**Background:**

Clear cell renal cell carcinoma (CCRCC) is the commonest form of kidney cancer. Up to 91% have biallelic inactivation of *VHL,* resulting in stabilisation of HIF-*α* subunits. Factor inhibiting HIF-1 is an enzyme that hydroxylates HIF-*α* subunits and prevents recruitment of the co-activator CBP/P300. An important question is whether FIH-1 controls HIF activity in CCRCC.

**Methods:**

Human VHL defective CCRCC lines RCC10, RCC4 and 786–O were used to determine the role of FIH-1 in modulating HIF activity, using small interfering RNA knockdown, retroviral gene expression, quantitative RT–PCR, western blot analysis, Annexin V and propidium iodide labelling.

**Results:**

Although it was previously suggested that FIH-1 is suppressed in CCRCC, we found that FIH-1 mRNA and protein are actually present at similar levels in CCRCC and normal kidney. The FIH-1 inhibition or knockdown in the VHL defective CCRCC lines RCC10 and RCC4 (which express both HIF-1*α* and HIF-2*α*) resulted in increased expression of HIF target genes. In the 786-O CCRCC cell line, which expresses only HIF-2*α*, FIH-1 attenuation showed no significant effect on expression of these genes; introduction of HIF-1*α* resulted in sensitivity of HIF targets to FIH-1 knockdown. In RCC4 and RCC10, knockdown of FIH-1 increased apoptosis. Suppressing HIF-1*α* expression in RCC10 prevented FIH-1 knockdown from increasing apoptosis.

**Conclusion:**

Our results support a unifying model in which HIF-1*α* has a tumour suppressor action in CCRCC, held in check by FIH-1. Inhibiting FIH-1 in CCRCC could be used to bias the HIF response towards HIF-1*α* and decrease tumour cell viability.

Clear cell renal cell carcinoma (CCRCC) often presents late and remains a significant cause of morbidity and mortality. Recently there have been advances in identifying signalling pathways that are activated in CCRCC, and the introduction of treatments targeting these pathways has shown useful activity in patients with the disease (Linehan *et al*, 2009). At a genetic level, biallelic inactivation of *VHL* occurs in the majority of CCRCC, and also underlies the tumours that develop in families with von Hippel–Lindau disease caused by a germline mutation in *VHL*. Re-expression of VHL efficiently suppresses tumour growth of CCRCC in xenograft assays, establishing that *VHL* acts as a gatekeeper tumour suppressor gene in the renal epithelium (Iliopoulos *et al*, 1995). However, understanding of the mechanism(s) by which VHL does this is challenging because it is a multifunctional protein (Frew and Krek, 2008). The best characterised function of *VHL* is its role as a negative regulator of hypoxia-inducible factor (HIF), through oxygen-dependent degradation of the HIF-*α* subunit, of which there are two isoforms HIF-1*α* and HIF-2*α* (Maxwell *et al*, 1999). Although it is likely that other actions of VHL contribute to its tumour suppressor action in the kidney, activation of HIF (and more specifically HIF-2*α*) has been shown to be necessary and sufficient for growth of VHL defective CCRCC cells in xenograft assays (Kondo *et al*, 2002, 2003). The HIF activation has a range of effects, which could contribute to tumour progression, including enhancing glucose uptake, and increasing expression of glycolytic enzymes and angiogenic mediators (Semenza, 2007). When VHL is present, HIF activation is dramatically downregulated in the presence of oxygen through oxygen-dependent enzymatic hydroxylation of specific prolyl residues by the prolyl hydroxylase domain (PHD) enzymes in the central part of HIF-*α* subunits, which leads to capture by VHL and ubiquitylation (Epstein *et al*, 2001). In VHL defective cells, HIF-*α* subunits are stable in the presence of oxygen (Maxwell *et al*, 1999). Studies in mice and humans have established that VHL loss-of-function alone is not sufficient for tumourigenesis (Mandriota *et al*, 2002; Rankin *et al*, 2006; Frew *et al*, 2008). The additional events that are required for tumour development are incompletely understood, but there is evolution from exclusive HIF-1*α* expression in normal renal epithelium and very early lesions to a predominant or exclusive HIF-2*α* response in tumours, which is likely to be important (Raval *et al*, 2005).

Co-activator recruitment by HIF-*α* subunits is regulated by oxygen via FIH-1. This hydroxylates a conserved asparagine residue (Asn 803 in human HIF-1*α*) within the C-terminal transactivation domain (CTAD) of HIF-*α*, thereby preventing binding of the co-factor p300 and inhibiting HIF transcriptional activation (Freedman *et al*, 2002; Hewitson *et al*, 2002; Lando *et al*, 2002a, 2002b; Elkins *et al*, 2003). The role of FIH-1 in regulating the HIF response has been less extensively investigated than that of the PHD enzymes and VHL, but it is established that attenuating FIH-1 increases expression of HIF target genes across a wide range of oxygen tensions (Stolze *et al*, 2004). Importantly, it has recently been established that FIH-1 hydroxylates ankyrin repeats in other proteins besides HIF-*α* subunits (Cockman *et al*, 2006; Linke *et al*, 2007).

Here we investigate the role of FIH-1 in modulating HIF activity in VHL defective CCRCC. Previous studies of two renal cancer cell lines, A498 and 786-O, suggested that FIH-1 expression was specifically repressed by a mechanism involving phosphatidylinositol 3-kinase (PI3K) and the atypical protein kinase C, PKC*ζ* (Datta *et al*, 2004; Li *et al*, 2007). Importantly, we found that FIH-1 is in fact present in CCRCC at similar levels to normal kidney. Further, we show that FIH-1 modulates HIF activity in VHL defective CCRCC lines that contain HIF-1*α* and HIF-2*α*, and that inhibiting FIH-1 decreases expansion of these cells in culture and increases apoptosis, in a HIF-1*α*-dependent manner.

## Materials and methods

### Cell culture, chemicals, and antibodies

Cell lines RCC10, RCC4 and 786-O, and RCC10 stable transfectants with wild-type pVHL have been described previously (Maxwell *et al*, 1999; Krieg *et al*, 2000). Cells were cultured in RPMI 1640 (Life Technologies, Gaithersburg, MD, USA) supplemented with penicillin/streptomycin, glutamine, and 10% fetal bovine serum. Cultures were incubated at 37°C in humidified air with 5% CO_2_.

Dimethyloxalylglycine (DMOG) was purchased from Frontier Scientific (Logan, UT, USA). For hypoxic experiments, cells were exposed to 0.1 and 1% oxygen for the indicated times using either a hypoxic workstation (INVIVO_2_ 100, Ruskinn, Leeds, UK) or a hypoxic incubator Galaxy R (Biotech, Palo Alto, CA, USA).

The antibodies used were as follows: FIH-1 (Novus biologicals, Littleton, CO, USA), HIF-1*α* (clone 54; Transduction Labs, Lexington, KY, USA), HIF-2*α* (p190b; Cancer Research UK, London, UK), GLUT1 and *β*-actin (Abcam, Cambridge, UK), α-tubulin (Sigma-Aldrich, Poole, UK), Pk-tag (AbD, Serotec, UK).

### Clinical material

The study was approved by the Hammersmith and Queen Charlotte's and Chelsea Research Ethics Committee (2002/6486). Following informed consent, samples of uninvolved kidney tissue and tumour were snap frozen in the operating theatre and stored at −80°C.

### Immunoblotting

Tissues and cells were homogenised in protein extraction buffer as described (Wiesener *et al*, 1998). Immunoblots were visualised with enhanced chemiluminescence (ECL) or ECL Plus (Amersham, Arlington Heights, IL, USA).

### Real-time reverse transcription-PCR

Total cellular RNA from cells and tissues was isolated using RNA Bee (Biogenesis, Poole, UK), according to the manufacturer's instructions. Total RNA (2 *μ*g per 20 *μ*l reaction) was retrotranscribed using an avian myeloblastosis virus retrotranscription kit (Roche, Indianapolis, IN, USA). PCR was carried out using an Opticon 2 machine (MJ Research, Waltham, MA, USA). Analysis of each experimental sample was in duplicate or triplicate. All real-time reverse transcription-PCR (RT–PCR) data are given as a value normalised to the level of *β-actin* expression in the same retrotranscription. For tumour samples, values were normalised to the level of 18S expression. The *β-actin* expression was not significantly altered by hypoxia, or DMOG.

*β-Actin*, 18S, glucose transporter 1 (*GLUT1*), BCL2/adenovirus E1B 19 kDa interacting protein 3 (*BNIP3*), factor inhibiting hypoxia-inducible factor (*FIH-1*), *PHD3* and vascular endothelial growth factor (*VEGF*) mRNA were measured using SYBR Green (ABgene, Epsom, UK) and the following primers:

*β-Actin*, 5′-CCCAGAGCAAGAGAGG-3′ (forward) and 5′-GTCCAGACGCAGGATG-3′ (reverse); 18S, 5′-CGCCGCTAGAGGTGAAATTC-3′ (forward) and 5′-TTGGCAAATGCTTTCGCTC-3′ (reverse); *GLUT1*, 5′-TGGCATGGCGGGTTGT-3′ (forward) and 5′-CCAGGGTAGCTGCTCCAGC-3′ (reverse); *PHD3*, 5′-GATGCTGAAGAAAGGGC-3′ (forward) and 5′-CTGGCAAAGAGAGTATCTG-3′ (reverse); *VEGF*, 5′-TGCCAAGTGGTCCCAG-3′ (forward) and 5′-GTGAGGTTTGATCCGC-3′ (reverse); *BNIP3*, 5′-GATATGGGATTGGTCAAGTCGG-3′ (forward) and 5′-CGCTCGTGTTCCTCATGCT-3′ (reverse); *FIH-1*, 5′-AAAATGTGGTTGGTTACGAAACAG-3′ (forward) and 5′-GACTCTATGTGATGCCACCAGTACA-3′ (reverse).

### Small interfering (si)RNA transfections

Sequences for siRNA targeting FIH-1 were generated using Target Finder (Ambion Bioscience, Austin, TX, USA) and purchased from Eurogentec (Southampton, UK). Transfections were performed in p60 culture dishes using LipofectAMINE 2000 (Invitrogen, San Diego, CA, USA) with siRNA oligos at a concentration of 50 nmol l^−1^. Cells were transfected as a pool, and after 15–20 h, were divided onto six-well dishes. Cells were analysed 2 days after transfection.

Small interfering RNA oligo sequences were as follows: **FIH #1**: F-5′-AUGAGGAGCCUGUGGUGCUdTdT-3′ R-5′-AGCACCACAGGCUCCUCAUdTdT-3′ **FIH #2**: F-5′-GAUGCUUGGAGAGGCCUUGdTdT-3′ R-5′-CCAAGGCCUCUCCAAGCAUdTdT-3′ **HIF-1***α*: F-5′-CUGAUGACCAGCAACUUGAdTdT-3′ R-5′-UCAAGUUGCUGGUCAUCAGdTdT-3′ **HIF-2***α*: F-5′-CAGCAUCUUUGAUAGCAGUdTdT-3′ R-5′-ACUGCUAUCAAAGAUGCUGdTdT-3′ **Firefly Luciferase (LUC)**: F-5′-CGUACGCGGAAUACUUCGAdTdT-3′ R-5′-AAGCUAAAGGUACACAAUUdTdT-3′.

### Lentiviral short hairpin RNA (shRNA) transfections

The RCC10 cells were plated at a density of 4 × 10^4^ per well in 24-well plates and cultured in complete media for 24 h. The following day, fresh media, supplemented with 6 *μ*g ml^−1^ polybrene (Sigma-Aldrich), were applied and the cells were transfected with lentiviral particles targeting HIF-1*α* (Sigma-Aldrich, MISSION shRNA, NM_001530, clone ID TRCN0000003810) or luciferase (Sigma-Aldrich, vector ID SHC007V, F-5′-CCGGCGCTGAGTACTTCGAAATGTCCTC-3′ R-5′-GAGGACATTTCGAAGTACTCAGCGTTTTT3′) at a multiplicity of infection of five. The following day fresh medium was added and 48 h post infection complete medium containing 2 *μ*g ml^−1^ puromycin was applied to select for transfected cells.

### Infection of HIF and FIH-1 retroviral vectors

Viral supernatants were prepared by transfecting the Phoenix packaging cell line (Orbigen, San Diego, CA, USA) using LipofectAMINE 2000. After the initial transfection, Phoenix cells were grown at 32°C. The supernatant was collected and filtered (0.45 *μ*m), then supplemented with 0.25-volume fresh medium with 7.5 *μ*g ml^−1^ polybrene (Sigma-Aldrich, Poole, UK), and added to cells that had been plated the day before on p100 dishes at 30–40% confluence. After 20 h, cells were washed, and fresh media were added for 20 h before performing a second round of infection.

An active form of HIF-1*α* carrying the substitutions P402A and P564A, which is resistant to hydroxylation by PHD enzymes, was cloned into pBMNz-HIF1*α*-neo (Raval *et al*, 2005). Following infection as described, 786-O cells were selected with G418.

### Retroviral vectors of pFIH-1

The coding sequence for human FIH-1 with and without a C terminal Pk tag (V5 epitope from paramyxovirus) was inserted into pCMVR-Neo using standard manoeuvres. Following infection with retroviruses, cells were selected with G418.

### Cell proliferation and apoptosis assays

Cell culture expansion was measured over a period of 3 days by MTT assay (3-(4,5-dimethylthiazol-2yl)-2,5-diphenyltetrazolium bromide; Sigma-Aldrich). Cells were transfected as described above and after 15–20 h, were plated at a density of 3 × 10^3^ cells per well into a 24-well cell tissue culture plate. After 24, 48 and 72 h, 50 *μ*l of 5 mg ml^−1^ MTT solution were added to the cell cultures in 0.5 ml of medium. After 4 h, media were removed and precipitated formazan crystals formed in viable cells were solubilised with 200 *μ*l of isopropanol-triton (0.1%). Product formation was quantified by absorbance at 550 nm.

Cell culture expansion was also assessed by manual counting. Transfected cells were plated at a density of 2 × 10^4^ cells per well into a six-well tissue culture plate, or 1 × 10^4^ cells per well into a 24-well tissue culture plate and viable cells were counted using a hemocytometer after trypan blue staining.

Apoptosis of siRNA-transfected cells was measured by the Annexin-V-FITC Detection Kit I (BD Biosciences, Oxford, UK) according to the manufacturer's instructions. After staining, cells were analysed on a Becton Dickinson FACS Caliber flow cytometer with CellQuest software (BD Biosciences).

Apoptosis was also measured using the Cell Death Detection ELISA Plus kit (Roche, Burgess Hill, UK). Cell pellets of transfected cells were placed into 200 *μ*l of lysis buffer provided by the manufacturer for 30 min and centrifuged. Aliquots of the supernatant (20 *μ*l) were used in an ELISA with anti-DNA and anti-histone antibodies to detect the presence of cytoplasmic nucleosomes.

### Statistical analysis

Data are presented as the mean (±s.e.m.) of three independent experiments. ANOVA or Student's *t*-test were used to evaluate differences and the level of statistical significance is indicated by the use of asterisks in the figures: ^*^P<0.05, ^**^P<0.01, ^***^P<0.001.

## Results

### Factor inhibiting HIF-1 is expressed in renal cancer

**Figure 1:**
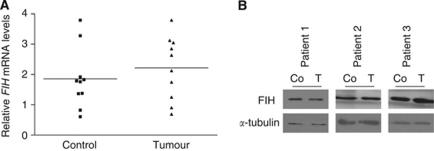
The FIH-1 is expressed at a similar level in renal cancer and uninvolved kidney. (**A**) The *FIH-1* mRNA levels in renal cancer samples and uninvolved kidney from the same patients. *n*=10. (**B**) Representative immunoblots showing FIH-1 protein in renal cancer samples and uninvolved kidney from the same patients.

The expression of FIH-1 in CCRCC has not been directly examined to our knowledge. We therefore analysed expression of FIH-1 in CCRCC samples and adjacent uninvolved kidney samples from the same patient. Specimens were obtained at the time of nephrectomy for CCRCC. Although the levels detected were variable, Figure 1 shows that there was no significant difference in *FIH-1* mRNA or protein levels between tumour specimens and adjacent kidney (Figures 1A and B). As expected for cancers where the HIF pathway is constitutively activated, *GLUT1* mRNA levels were increased in the tumour samples (data not shown).

### Factor inhibiting HIF-1 functions in VHL defective CCRCC cell lines

**Figure 2:**
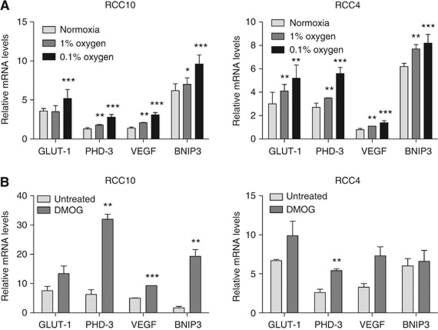
Effect of hypoxia and DMOG on gene expression in RCC10 and RCC4 cells. (**A**) Expression of the indicated HIF target genes was examined by qRT–PCR in cells that were cultured under standard conditions, 1% oxygen or 0.1% oxygen for 16 h. (**B**) Expression of HIF target genes following exposure to the 2-oxoglutarate analogue DMOG (500 *μ*M, 16 h). Data are presented as the mean of three independent experiments. (^*^P<0.05, ^**^P<0.01, ^***^P<0.001).

The presence of equivalent amounts of FIH-1 in tumour and adjacent kidney suggested that FIH-1 may actually be reducing HIF activity in this setting; HIF-*α* subunits would be abnormally stabilised (because VHL is absent), but HIF transcriptional activity would be limited by the action of FIH-1 in the presence of oxygen, which would hydroxylate the CTAD and reduce co-activator recruitment. To examine this we first exposed VHL defective RCC10 cells to reduced oxygen, as a means to reduce FIH-1 enzymatic activity. As Figure 2A shows, we found increased mRNA levels of four well-characterised HIF-*α* target genes *GLUT1*, *PHD3*, *VEGF* and *BNIP3* in RCC10 and RCC4 at 0.1 and 1% oxygen (Figure 2A). Expression of each of these genes was further increased in hypoxia from the high levels observed in the absence of VHL. Importantly, similar results were obtained in a second *VHL* defective cell line, RCC4 (Figure 2A). As reducing oxygenation could have other effects besides attenuating FIH-1 activity, we used DMOG, a small molecule inhibitor that is an analogue of the co-substrate 2-oxogluarate as an alternative method of inhibiting FIH-1. Figure 2B shows that treatment of CCRCC cell lines with DMOG increases expression of HIF target genes.

**Figure 3:**
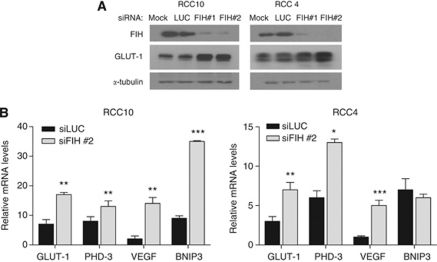
The siRNA knockdown of FIH-1 in renal cancer cell lines increases expression of HIF target genes. (**A**) The RCC4 and RCC10 cells were treated with siRNA for FIH-1 or luciferase (control). The immunoblot shows FIH-1 was decreased at the protein level and that this was associated with an increase in expression of the HIF target gene GLUT1. (**B**) The effect of FIH-1 knockdown on expression of the indicated HIF target genes in RCC10 and RCC4 cells. Data are presented as the mean of three independent experiments. (^*^P<0.05, ^**^P<0.01, ^***^P<0.001).

These results would be consistent with FIH-1 exerting a negative effect on HIF activation in these VHL defective cell lines. However hypoxia and DMOG are not specific inhibitors of FIH-1; they will inhibit other 2-oxoglutarate-dependent oxygenases including the PHD enzymes (Epstein *et al*, 2001). In the absence of VHL, prolyl hydroxylation of HIF-1*α* and HIF-2*α* by the PHD enzymes has been reported to decrease transactivation by the *N*-terminal transactivation domain (NTAD) (To and Huang, 2005), providing a potential mechanism by which hypoxia and DMOG would increase expression of HIF targets in a manner independent of FIH-1 and CTAD activity. To directly examine whether FIH-1 inhibits HIF activity we used RNA interference (RNAi). By using either of two different non-overlapping siRNA sequences independently, we achieved a significant reduction of FIH-1 at the mRNA (>70% attenuation) and protein level. The FIH-1 knockdown resulted in a significant increase in HIF target gene mRNA levels as well as GLUT1 protein levels (Figures 3A and B). Taken together, these results provide clear evidence that FIH-1 is acting to reduce expression of HIF target genes in RCC10 and RCC4 cells under normoxic conditions.

### Increasing FIH-1 expression has little effect on the expression of HIF target genes

**Figure 4:**
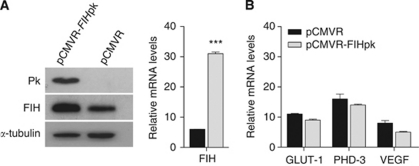
Overexpressing FIH-1 in RCC10 cells has little effect on expression of HIF target genes. (**A**) The RCC10 cells were infected with pCMVR-FIHPk, which encodes FIH-1 with a Pk tag in or empty vector followed by selection with G418 (1.5 mg ml^−1^). The immunoblot shows a signal at ∼40 kDa using a Pk antibody, confirming the expression of tagged FIH-1 protein. (**B**) Results of qRT–PCR analysis of gene expression showing an ∼six-fold increase in FIH-1 mRNA. The mRNA levels of the indicated HIF targets were not significantly affected by the overexpression of FIH-1 in RCC10. Data are presented as the mean of three independent experiments. (^***^P<0.001)

The inhibition of HIF transcriptional activity by FIH-1 in RCC10 and RCC4 cells is clearly incomplete as HIF exerts potent effects on gene expression in these cells, as demonstrated by the effects of siRNA for HIF-*α* subunits (Raval *et al*, 2005). One explanation for this incomplete inhibition would be that the amount of FIH-1 enzyme in these cells is insufficient to achieve maximal downregulation of HIF. We therefore examined the effect of increasing expression of FIH-1. A retroviral vector containing cDNA encoding for FIH-1 was prepared and used to infect RCC10 cells. The FIH-1 sequence was tagged with the Pk epitope to allow detection of the exogenous FIH-1 (Figure 4A). Analysis of mRNA levels showed a ∼six-fold increase in FIH-1 mRNA when compared with levels in RCC10 cells infected with an empty vector (Figure 4B). In case the Pk tag might reduce enzymatic activity, we also performed this experiment with untagged FIH-1, with similar results (data not shown). This lack of effect of augmenting FIH-1 contrasts with the effect of introduction of VHL, which leads to marked suppression of HIF target genes (Maxwell *et al*, 1999). This implies that a substantial proportion of HIF activity is resistant to the action of FIH-1; probably this involves transactivation mediated by the NTAD of HIF-*α* subunits, which is not regulated by FIH-1.

### Attenuating FIH-1 does not reduce HIF target gene expression in 786-O cells, which only express HIF-2*α*

**Figure 5:**
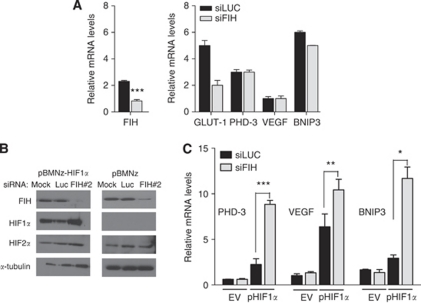
Introducing HIF-1*α* in 786-O cells reveals the ability of FIH-1 to regulate HIF activity. (**A**) The 786-O cells were treated with siRNA for FIH-1 or control. No increases in HIF target gene mRNA were observed, contrasting with results from RCC10 and RCC4 (Figure 2). Data are presented as the mean of three independent experiments. (**B**) The 786-O cells were infected with retrovirus-encoding HIF-1*α* in which the two prolyl residues that are targets for hydroxylation were mutated (left panels), or empty vector (right panels). Following selection with G418, cells were treated with luciferase control siRNA or FIH-1 siRNA. Immunoblots show expression of FIH-1, HIF-1*α* and HIF-2*α*. (**C**) Analysis of expression of the indicated HIF target genes. Inhibition of FIH-1 in cells infected with pBMNz empty vector (EV) does not increase HIF target gene expression. An increase in expression of HIF target genes following FIH-1 inhibition is seen following introduction of HIF-1*α*. Data are presented as the mean of three independent experiments. (^*^P<0.05, ^**^P<0.01, ^***^P<0.001).

To investigate this further we examined the effect of FIH-1 knockdown in another well-characterised VHL defective cell line, 786-O, which expresses HIF-2*α*, but not HIF-1*α* (Maxwell *et al*, 1999). We found that FIH-1 siRNA has no effect on HIF target gene expression in 786-O cells (Figure 5A). This contrasts with our observations in RCC10 and RCC4 cells, but is in line with a previous study (Datta *et al*, 2004). Possible explanations for this would be either that FIH-1 was inactive in these cells (as was suggested in the previous study), or that the HIF-2*α* they contain is not susceptible to inactivation by FIH-1. To distinguish these possibilities, we expressed HIF-1*α* in 786-O cells, and then performed RNAi against FIH-1. Western blot analysis confirmed exclusive expression of the HIF-2*α* isoform in a pool of parental 786-O cells infected with empty vector (pBMNz) and expression of HIF-1*α* in cells infected with pBMNz-HIF-1*α* (Figure 5B). Using siRNA, we attenuated FIH-1 in both pools of 786-O cells (Figure 5B). In the pBMNz transfected pool, in which there is exclusive expression of HIF-2*α*, attenuation of FIH-1 did not affect HIF target gene expression. In contrast, in pBMNz-HIF-1*α*, FIH-1 attenuation augmented HIF target gene levels of *PHD3*, *VEGF* and the pro-apoptotic gene *BNIP3* (Figure 5C). These results show that active FIH-1 is present in 786-O cells, and introducing HIF-1*α* can reveal this activity. Furthermore, HIF-2*α* (at least in 786-O cells) is insensitive to inactivation by FIH-1.

### Attenuating FIH-1 reduces growth of renal cancer cells expressing HIF-1*α* and induces apoptosis

**Figure 6:**
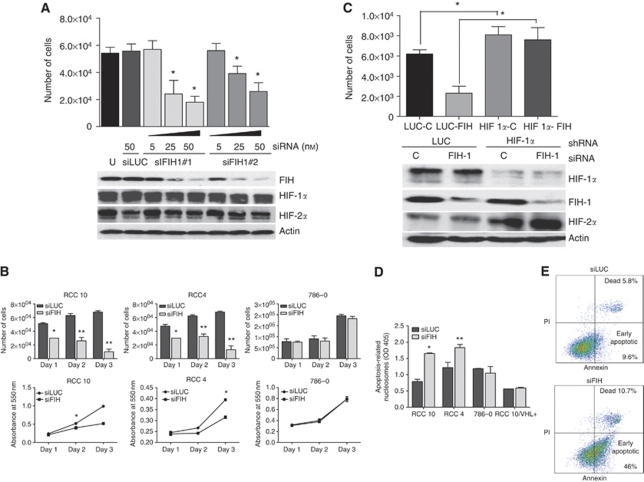
Effects of FIH-1 knockdown on population expansion of CCRCC cell lines. (**A**) The RCC10 cells were cultured in parallel and either untreated, treated with the indicated concentrations of two different siRNAs targeting FIH-1, or control siRNA at 50 nM. After 72 h, cell numbers were counted (^*^P<0.05). Immunoblot shows expression of FIH-1, HIF-1*α*, and HIF-2*α*. (**B**) Cultures of RCC10, RCC4 and 786-O, which were treated with FIH-1 siRNA or control siRNA were assessed by counting the number of viable cells at the indicated timepoints (top panel). Cultures of RCC10, RCC4 and 786-O were treated with FIH-1 siRNA or control siRNA and assessed by MTT assay (lower panel). Data are presented as the mean of three independent experiments (^*^P<0.05, ^**^P<0.01). (**C**) The RCC10 cells were infected with shRNA vectors for luciferase (control) or HIF-1*α*. Following selection with G418 they were treated either with control or FIH-1 siRNA and the number of cells was counted 72 h later. The effect of FIH-1 knockdown on cell number was abrogated by knockdown of HIF-1*α*. Note also that HIF-1*α* knockdown significantly increased cell numbers compared with control (compare bars 1 and 3). Immunoblots show expression of HIF-1*α*, HIF-2*α* and FIH-1. (**D**) Cytoplasmic histone-associated DNA fragments were assessed in the indicated cell lines following treatment with FIH-1 siRNA or control. Increased cell death occurred in RCC10 and RCC4 but not in 786-O cells (which express only HIF-2*α*) or RCC10/VHL in which re-expression of VHL restores HIF-*α* to basal levels (+ve). Data are presented as the mean of three independent experiments (^*^P<0.05, ^**^P<0.01). (**E**) Effect of FIH-1 knockdown on apoptosis in RCC10 and 786-O cells. Cells were transfected with siLUC or siFIH and analysed for apoptosis using the BD Pharmingen FITC Annexin V apoptosis detection kit. Plots are representative of two independent experiments.

Previously it has been shown that HIF-1*α* has anti-proliferative effects in VHL defective renal cancer cells (Raval *et al*, 2005). This raises the interesting possibility that in cells that lack VHL and express HIF-1*α*, FIH-1 may favour tumour growth by decreasing the anti-proliferative consequences of HIF-1*α* activation and shifting the balance of HIF activation towards HIF-2*α*. To test this, we examined the effect of FIH-1 siRNA on cell population expansion and apoptosis of RCC10 and RCC4 cell cultures. Knockdown of FIH-1 significantly reduced expansion of RCC10 cell cultures using either of the two siRNAs (Figure 6A). The RCC4 and RCC10 cells showed reduced population expansion as assessed by counting the number of viable cells or by MTT assays (Figure 6B). In contrast, population expansion of 786-O cells that do not express HIF-1*α* was not reduced. This suggested that the effect of FIH-1 knockdown on population expansion might be mediated via increasing the activity of HIF-1*α*. To test this possibility, RCC10 were transfected with shRNA targeting HIF-1*α* before FIH-1 knockdown. This prevented the effect of FIH-1 knockdown on population expansion (Figure 6C). Interestingly, suppression of HIF-1*α* expression resulted in modest, but statistically significant, increase in cell numbers in comparison with control, consistent with HIF-1*α* suppressing proliferation and/or enhancing cell death.

To investigate the mechanism(s) by which cell numbers were decreased, we assayed cytoplasmic histone-associated DNA fragments to assess apoptotic cell death (Figure 6D). This was increased by attenuating FIH-1 in VHL defective RCC10 and RCC4 cells. However, no significant increase was observed in 786-O cells (which express only HIF-2*α*) or in RCC10 cells in which VHL was stably expressed, resulting in suppression of HIF-1*α* (and HIF-2*α*). Independent evidence for increased apoptosis was provided by flow cytometry analysis (Figure 6E). The FIH-1 knockdown in RCC10 cells resulted in an increase in early apoptotic (Annexin-V positive) cells, compared with the control siLUC transfection (46.0 *vs* 9.6%). Taken together, the results are consistent with FIH-1 decreasing apoptosis through a decrease in the activity of HIF-1*α*. The 786-O cells showed a much less marked effect, but interestingly there was some increase in early apoptotic cells, on FIH-1 knockdown (16.59% with siRNA for FIH *vs* 5.7% in controls) (Data not shown). This may reflect a HIF-*α*, independent action of FIH-1 mediated by one of a number of other identified substrates, which includes components of the Notch or NF*κ*B pathways (Cockman *et al*, 2006; Coleman *et al*, 2007; Zheng *et al*, 2008).

## Discussion

The major findings of this study are that FIH-1 is present at similar levels in normal kidney and CCRCC, that FIH-1 inhibition in CCRCC cells can increase expression of HIF targets, and that inhibiting FIH-1 can increase apoptosis in these cells. It is noteworthy that FIH-1 expression is maintained in CCRCC compared with normal kidney, as it was previously suggested that an important step in evolution to CCRCC following the loss of VHL function was suppression of FIH-1. In that model, suppression of FIH-1 was considered necessary to achieve HIF activation. Our study shows that FIH-1 is present and active in CCRCC, but it only partially inactivates HIF. This is consistent with the fact that biallelic inactivation of *vhl* in mouse and human renal epithelium (and other cell types) is associated with marked activation of HIF target genes, even though FIH-1 has not been inactivated (Mandriota *et al*, 2002; Rankin *et al*, 2006).

Evidence for the model in which suppression of FIH-1 was a pivotal aspect of CCRCC included the fact that decreasing FIH-1 did not influence HIF activity in 786-O cells. Our study confirms this observation, but implies a different mechanism in which FIH-1 is present but the HIF-2*α* in these cells is resistant to its action. In the previous studies it was suggested that FIH-1 suppression was achieved in CCRCC via PKC*ζ* suppressing FIH-1 at the level of transcription. Our finding that FIH-1 expression is similar in normal kidney and CCRCC implies that any negative effect of PKC*ζ* is likely to be present in normal renal epithelium as well as CCRCC. This is supported by the fact that similar suppression of FIH-1 via PKC was also observed in HEK 293 cells, a non-malignant renal cell line (Li *et al*, 2007).

Although we show that FIH-1 is present in CCRCC, it is striking that its action on HIF ranges from partial suppression (RCC10 and RCC4 cells) to no significant effect (in 786-O cells). Several factors are likely to contribute. First, the amount of FIH-1 available may be insufficient—for example, it may be sequestered through binding to ankyrin repeat domains. However, it is notable that overexpression of FIH-1 had only a minor effect on HIF activity. Second, a substantial proportion of transactivation by HIF-*α* is independent of the interaction with the CH1 pocket of CBP/P300 (Kasper *et al*, 2005), this would be predicted to be insensitive to FIH-1 and will include the action of the NTAD. Third, a subset of HIF-*α* subunits may be resistant to the action of FIH-1. As HIF-1*α* is known to be a substantially better substrate for FIH-1 than HIF-2*α* (Bracken *et al*, 2006) it is likely that this resistant fraction is predominantly HIF-2*α*. This is further supported by data that mutating the FIH-1 target residue in HIF-2*α* did not increase its effect on HIF target genes or tumour growth in CCRCC cells, whereas substituting the equivalent residue in HIF-1*α* had a major effect (Yan *et al*, 2007). This is consistent with 786-O cells not showing evidence of increased HIF activity following FIH-1 knockdown, as they only contain HIF-2*α*. In addition, it is clear that a proportion of HIF-1*α* in RCC10 and RCC4 is not inactivated by FIH-1, as RNAi for HIF-1*α* in these cells has potent effects on gene expression and epithelial behaviour (Esteban *et al*, 2006). A possibility that merits further investigation is that a post-translational modification renders a proportion of HIF-1*α* resistant to the action of FIH-1. Reported modifications of HIF-*α* that could be candidates include phosphorylation, acetylation and sumoylation. An important candidate modification is phosphorylation of Thr-796 in HIF-1*α*, which can prevent hydroxylation by FIH-1 (Lancaster *et al*, 2004).

Regardless of the mechanism(s) by which a substantial proportion of HIF activity in CCRCC is resistant to FIH-1, we show that FIH-1 is protecting RCC10 cells from apoptosis and this is mediated by its effect on HIF-1*α*. That increased activity of HIF is associated with increased apoptosis may appear counter-intuitive, in view of the evidence that suppressing of HIF is a major aspect of VHL's action as a tumour suppressor in the kidney. On the other hand, it is well recognised that although many of HIF-1*α*'s actions promote tumour growth it can also promote cell cycle arrest and apoptosis. Activation of HIF-1*α* subunit increases expression of genes that are pro-apoptotic (Sowter *et al*, 2001; Greijer and van der Wall, 2004) and overexpression of HIF-1*α* has been shown to reduce growth of CCRCC cells as xenografts (Volm and Koomagi, 2000; Raval *et al*, 2005). Furthermore, foci of biallelic inactivation of VHL in human kidney, which show HIF-1*α* activation, are not associated with a net increase in proliferation (Mandriota *et al*, 2002). This suggests that evolution to CCRCC involves a number of additional steps, and there is evidence that a progressive increase in HIF-2*α* relative to HIF-1*α* is important. Consistent with this, in xenograft assays of CCRCC cells, active HIF-2*α* is both necessary and sufficient for tumour growth (Kondo *et al*, 2002, 2003), whereas active HIF-1*α* is insufficient (Maranchie *et al*, 2002). An attractive possibility is that by exerting more effect on HIF-1*α* than HIF-2*α*, FIH-1 contributes to VHL defective cells evading apoptosis. This is also consistent with the fact that mutations in FIH-1 have not been reported in CCRCC (Morris *et al*, 2004).

The intersection of the effects of VHL, HIF and FIH-1 on patterns of gene expression and on cell proliferation have recently been examined in murine embryonic fibroblasts with genetic deletions of each gene, and multiple combinations thereof (Zhang *et al*, 2010). In murine embryonic fibroblasts (MEFs) loss of FIH-1 was shown to have significant and complex effects on expression of HIF target genes in the absence of VHL under normoxic conditions. Loss of VHL in MEFs was associated with reduced plating efficiency and there was an additive negative effect of loss of FIH-1, consistent with the effects that we observe of FIH-1 knockdown on population expansion in CCRCC cells. Strikingly, removal of HIF-1*α* restored plating efficiency to that of controls.

The crystal structure of FIH-1 has been solved and there are prototype inhibitors implying it will be druggable (Banerji *et al*, 2005; McDonough *et al*, 2005; Moon *et al*, 2010). An important consideration is that other FIH-1 substrates have been identified besides HIF-1*α*. In particular, it has been shown that asparagine residues in ankyrin repeat domains are hydroxylated by FIH-1 including the intracellular domain (ICD) of the Notch receptor, the I*κ*B family of inhibitory proteins and tankyrase (Cockman *et al*, 2006, 2009; Coleman *et al*, 2007; Ferguson *et al*, 2007; Zheng *et al*, 2008). Therefore FIH-1 inhibition is likely to have wide-ranging effects that would have to be investigated before FIH-1 can be considered a suitable target for inhibition.

Useful insight into this is provided by the recently reported knockout mouse, which is viable but exhibits hypermetabolism (Zhang *et al*, 2010). Our study raises the possibility that FIH-1 would be a useful therapeutic target in clear cell renal carcinoma.
